# A Unique Case of Intra-Abdominal Diffuse Lymphangiomatosis Mimicking a Pseudomyxoma Peritonei

**DOI:** 10.70352/scrj.cr.24-0037

**Published:** 2025-02-21

**Authors:** Andreas R. R. Weiss, Georg F. Weber, Maximilian Brunner, Robert Grützmann, Abbas Agaimy, Christian Krautz

**Affiliations:** 1 Department of Surgery, Erlangen University Hospital, Comprehensive Cancer Center Erlangen-EMN, Friedrich-Alexander-University Erlangen -Nuremberg (FAU), Erlangen, Germany; 2 Bavarian Cancer Research Center (BZKF), Erlangen, Germany; 3 Institute of Pathology, Friedrich-Alexander-University Erlangen-Nürnberg, University Hospital, Erlangen, Germany

**Keywords:** diffuse lymphangioma, abdominal lymphangiomatosis, pseudomyxoma peritonei, cytoreductive surgery

## Abstract

**INTRODUCTION:**

Localized cystic lymphangiomas (CL) are rare benign tumors deriving from the lymphatic system. CL is diagnosed more frequently in children than in the adult population and, although commonly affecting the cervical and axillary regions, can develop in various parts of the body. Abdominal cystic lymphangioma (ACL) comprises less than 5% of all CL cases.

**CASE PRESENTATION:**

A 35-year-old female patient with a history of benign appendectomy in childhood was transferred to our tertiary center for the operative management of a suspected extensive pseudomyxoma peritonei (PMP). In accordance with the multidisciplinary team discussion, cytoreductive surgery with hyperthermic intraperitoneal chemotherapy was planned. Intraoperatively, a typical “jelly belly” with high disease burden throughout the abdominal cavity and the small pelvis was found. A multi-visceral resection with complete cytoreduction (CCR 0) was performed. The postoperative histopathological findings revealed a diffuse, partially cystic lymphangiomatosis involving the peritoneum extensively without evidence of PMP or malignancy.

**CONCLUSIONS:**

ACLs are uncommon in the adult population, and diffuse peritoneal involvement is even rarer. Surgical management with complete resection is the preferred treatment option. Other benign cysts, as well as infectious diseases and malignancy, should be considered during the preoperative workup.

## Abbreviations

ACL
: abdominal cystic lymphangioma
CA
: carbohydrate antigen
CCR 0
: complete cytoreduction
CEA
: carcinoembryonic antigen
CL
: cystic lymphangioma
CRS
: cytoreductive surgery
CT
: computed tomography
HIPEC
: hyperthermic intraperitoneal chemotherapy
MDT
: multidisciplinary team
PCI
: peritoneal carcinomatosis index
PMP
: pseudomyxoma peritonei

## INTRODUCTION

Cystic lymphangioma (CL) is a rare benign proliferative malformation of the lymphatic vascular system and is considered to be congenital in origin.^1,2)^ More than 80% of CL are diagnosed in children below the age of 1 year.^3)^ CL commonly affects the neck and axillary regions of the body, and they present as localized mass-forming lesions. Rarely, CL may present with extensive diffuse disease, including diffuse peritoneal lymphangiomatosis and kaposiform lymphangiomatosis, frequently associated with *NRAS* gene mutations.^4,5)^ Abdominal cystic lymphangioma (ACL) accounts for <5% of all CL cases.^6)^ In the current literature, only case reports and case series with a maximum of 32 patients can be found. ACL are detected most often in the small bowel mesentery, followed by the greater omentum, the mesocolon, and the retroperitoneum.^3)^ The clinical presentation seems to be highly variable. While some patients remain completely asymptomatic, others may present with an acute abdomen due to an intestinal volvulus, obstruction, or bleeding.^6–8)^ The following case report describes a unique case of an extensive diffuse intra-abdominal lymphangiomatosis that presented with typical features of a pseudomyxoma peritonei and was treated accordingly.

## CASE PRESENTATION

**Fig. 1:**
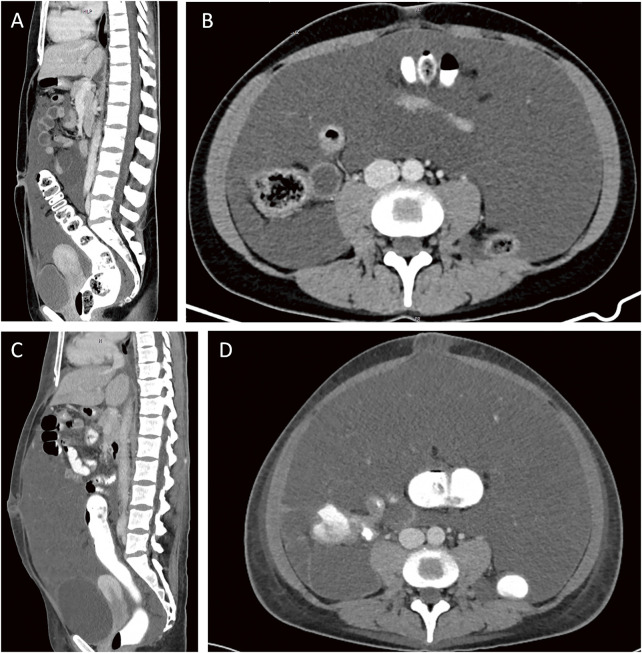
CT images 15 days (**A, B**) and 5 days (**C, D**) before surgery. CT, computed tomography

**Fig. 2:**
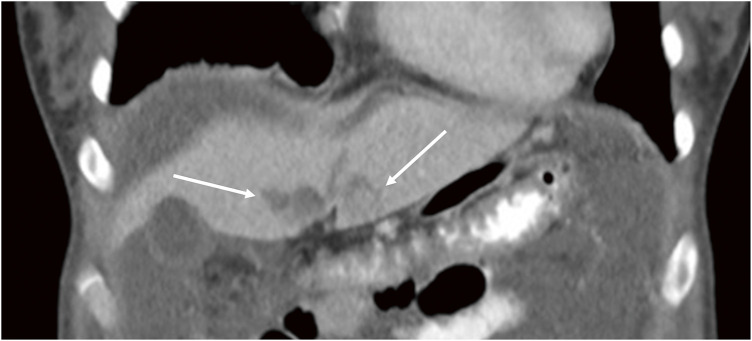
Scalloping of the liver surface (white arrows).

An otherwise fit and well 35-year-old female patient without any remarkable medical history apart from a benign appendicectomy for an ulcero-phlegmonous, purulent appendicitis 20 years ago presented to an external hospital with a 1-day history of increasing abdominal pain, vomiting, and fevers. The patient herself had noted an increase in the size of her belly over the last few years, but she has had no symptoms previously. In the emergency department, a pregnancy was ruled out. For further investigations, a computed tomography (CT) scan of the abdomen and pelvis was obtained, which demonstrated large amounts of ascites with dot-like calcifications and subtle septations that seemed to encase small bowel loops, ovaries, and uterus with some of the formations extending into the retroperitoneal space (**Figs. 1A**, **1B**). Furthermore, the ovaries appeared to be prominent, and the serum level of the tumor marker carbohydrate antigen 125 (CA-125) was elevated at 78.9 U/mL (normal range 0–35 U/mL). Carcinoembryonic antigen (CEA) and CA 19-9 were within normal limits. The case was presented at our cancer multidisciplinary team (MDT) meeting, and the findings were thought to be highly suspicious for ovarian cancer, with pseudomyxoma peritonei (PMP) being the differential diagnosis. However, a subsequent gynecological workup did not reveal any pathological findings in the genital tract. A further workup with an OGD and a colonoscopy was also unremarkable. Due to worsening symptoms, another CT scan of the patient’s abdomen was performed 10 days after the initial scan and showed a significant increase in the amount of ascites as well as visceral scalloping on the liver surface not previously seen on CT (**Figs. 1C**, **1D**; **Fig. 2**). Due to increasing inflammatory markers and a suspected spontaneous bacterial peritonitis an empiric antibiotic treatment with piperacillin/tazobactam was started. The case was re-discussed at our cancer MDT, where, as an ovarian primary had been ruled out, the findings were thought to be most in keeping with a PMP. The MDT recommended cytoreductive surgery (CRS) followed by hyperthermic intraperitoneal chemotherapy (HIPEC). For the surgery, the patient was transferred to our maximum care facility, and the preoperative workup was completed. On the day of admission, the inflammatory markers were still significantly elevated, with a CRP of 207 mg/L and a leukocytosis of 11.7 × 10³/μL, so the empiric antibiotic treatment was continued. However, the inflammatory markers remained unchanged over the next week, and the fevers, which peaked at 39°C, persisted. The patient had ongoing abdominal discomfort with intermittent crampy pain, nausea, and generalized abdominal tenderness but no frank peritonitis. By then, it was assumed that the pseudomyxoma itself was causing the symptoms, including the rise in the inflammatory markers as well as the ongoing fevers, so the surgery was planned and carried out 9 days after the patient’s admission to our hospital.

**Fig. 3:**
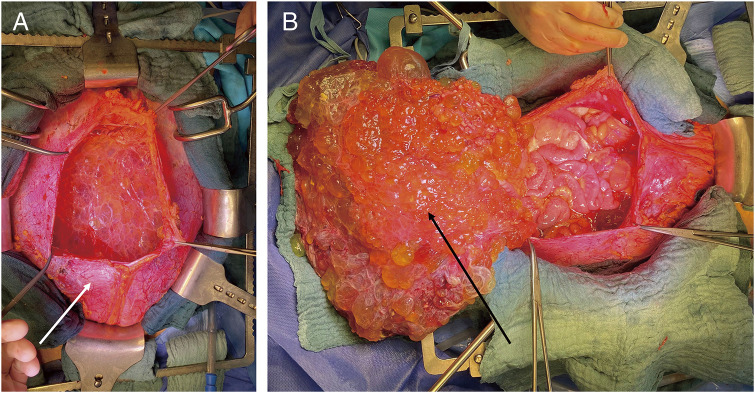
(**A)** Mobilized and opened peritoneal sac (white arrow). (**B**) Opened peritoneal sac with the omentum/omental cake (black arrow) lifted outside.

**Fig. 4:**
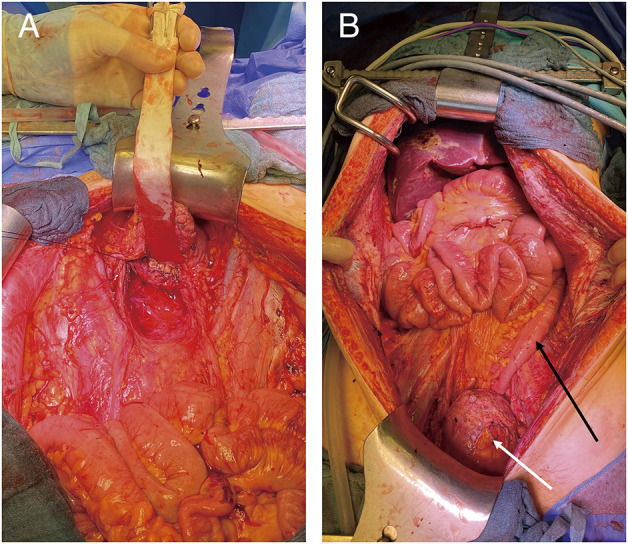
(**A**) View of the abdomen after complete cytoreduction, including near-total peritonectomy, low anterior resection, right hemicolectomy, jejunal segmental resection, supracervical hysterectomy, bilateral salpingo-oophorectomy, resection of extraperitoneal deposits in the small pelvis, and cholecystectomy (sling placed around left ureter). (**B**) View after reconstruction with jejuno-jejunostomy, ileo-colic anastomosis, and colo-rectal anastomosis. Black arrow = mobilized descending colon, white arrow = retrogradely filled urinary bladder

**Fig. 5:**
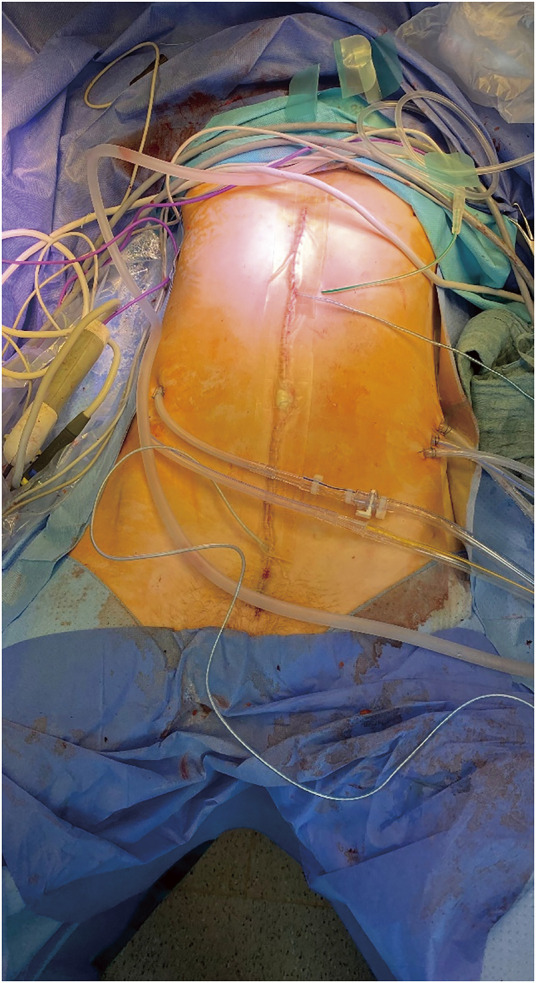
Abdomen with 5 large bore drains in situ during HIPEC. Two temperature electrodes and 1 subcutaneous redon drain in the midline. HIPEC, hyperthermic intraperitoneal chemotherapy

During the surgery, a median laparotomy from the xiphisternum to the pubic symphysis was performed, and the subcutaneous tissue and the fascia were divided. The bulging peritoneal sac was kept intact for as long as possible and the preparation was carried on extraperitoneally down into both flanks and into the retropubic space. After a near total mobilization of the entity of the peritoneal sac, the peritoneum was opened in the upper midline, and several liters of the jelly-like content was removed (**Fig. 3**). A thorough exploration of the abdominal cavity revealed an omental cake, a heavy disease burden with dense cystic implants on the bowel wall of the ileo-caecal region and on the mid jejunum as well as in the pelvis with significant affection of the rectum and the ovaries. Deposits were also found in the retroperitoneum and within the extraperitoneal space. Taken together, a peritoneal carcinomatosis index (PCI) of 39 was calculated. Subsequently, cytoreductive surgery with a near total peritonectomy, right hemicolectomy, low anterior resection, jejunal segmental resection, supracervical hysterectomy, bilateral salpingo-oophorectomy, cholecystectomy, and omentectomy was performed. The deposits on the liver capsule were surgically removed as far as possible, and the remnants were ablated with bipolar forceps. Further small deposits on the small and large bowel mesentery were resected and/or ablated with bipolar forceps, and deposits on the bladder wall, vaginal vault, and along the iliac vessels were surgically removed. A complete cytoreduction (CCR 0) was achieved (**Fig. 4A**). The intestinal passage was reconstructed with a double-layered end-to-end hand-sewn jejuno-jejunostomy, a double-layered end-to-end hand-sewn ileo-colic anastomosis, and an end-to-end stapled colorectal anastomosis (**Fig. 4B**). Five large bore drains (20F) were placed. The surgery took 7 h and 43 min. The estimated blood loss was 300 mL. Following the surgery, a HIPEC with 30 mg of Mitomycin C was performed on the operating table over 60 minutes (**Fig. 5**). The patient was extubated at the end of the case and transferred to our surgical intensive care unit. After an uneventful night, the patient was transferred to the normal ward the next day. Due to a symptomatic anemia with an Hb of 6.5 g/dL on the background of a significant preoperative anemia of 9.3 g/dL in combination with extensive generalized edema, 2 red blood cell units were transfused on that day, and a diuretic treatment was initiated. The further course was rather uneventful. Oral diet was recommenced on postoperative day 2 and only slowly increased over the next 5 days due to intermittent nausea. The generalized edema was significantly improving, so the diuretic medication was ceased. The antibiotic treatment was continued postoperatively for 8 more days and ceased after the inflammatory markers and clinical condition of the patient had significantly improved. Interestingly, there was no microbiological evidence of a bacterial infection within the abdomen. The patient was discharged on a regular diet without nausea on postoperative day 13.

**Fig. 6:**
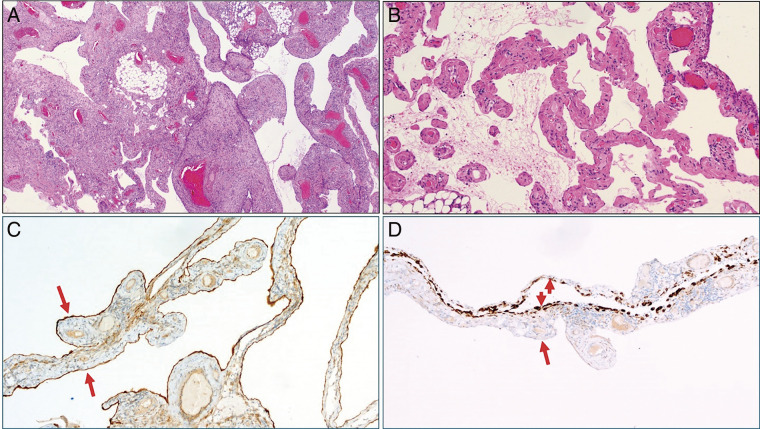
(**A**) Diffuse solid and cystic peritoneal thickening. (**B**) High power showing cystic spaces separated by thin peritoneal layers/lymphatic walls. (**C**) D2-40 (podoplanin) stains both sides of the thin walls (long arrows; one side corresponds to the mesothelial superficial covering and the other to the lymphatic endothelium lining the cyst lumens). (**D**) On the contrary, pankeratin is only expressed on the peritoneal side (short arrows) but was negative on the endothelial side of the cyst wall (long arrows), confirming lymphangiomatosis and excluding mesothelial inclusion cysts.

The histological findings did not show any evidence of PMP despite the typical clinical and intraoperative findings. Instead, the diagnosis of a partially cystic, diffuse peritoneal lymphangiomatosis was made (**Fig. 6**). Diffuse peritoneal inclusion cysts (so-called benign multi-cystic mesothelioma of the peritoneum) were excluded based on the immunophenotype of the cells lining the cysts (positive for both panendothelial markers and D2-40/podoplanin but negative for keratins and calretinin). In this particular case, the tumor/ACL presented as a mixture of cystic lesions of varying sizes and large amounts of viscous, jelly-like material as well as more liquid, ascites-like components (**Fig. 3**). There was no evidence for malignancy.

The patient presented to our emergency department 6.5 weeks after discharge with left flank pain over the previous 24 h and vomiting. A CT scan showed a mild to moderate dilation of the left renal pelvis with an obstruction at the level of the distal ureter due to a 3 mm ureteric stone as well as non-specific fluid collections around the liver hilum and in the gall bladder fossa. Inflammatory markers and serum creatinine were normal, and the patient was treated conservatively and discharged with oral pain medication. When reviewed 5 days later, the patient had fully recovered with no more complaints of flank pain or nausea and was discharged from our outpatient clinic. A follow-up with the urology department 2 weeks later revealed that the stone had passed as the dilatation of the left renal pelvis had resolved at the time.

## DISCUSSION

PMP and ACL both are rare entities with similar incidence rates of 3.2 and 4 per million, respectively.^9,10)^ However, ACL is diagnosed in children in 80% to 90% of cases, which makes PMP more common in the adult population.

The gold standard for the treatment of ACL is complete surgical resection. Asymptomatic patients may be followed by repeated imaging only, as some CLs have been described to regress spontaneously over time. Attempts were also made with aspiration of the cystic content, although this bears a high risk for recurrence. For unresectable lymphangioma, sclerotherapy with doxycycline or alcohol or radiotherapy has been applied with some success.^6,7,11)^

To our knowledge, this is the most extensive case of ACL that has been described in the literature to date.^7)^ The clinical presentation with key features like abdominal distension with a “jelly belly,” progressive intestinal obstruction, and abdominal pain mimicked the typical presentation of a patient with advanced PMP.^12,13)^ Cases of superinfected PMP have also been described, in which patients were reported to have fevers as well as raised inflammatory markers and—like our patient—a diffusely tender abdomen without peritonitis.^14)^ Fever due to an infected ACL was reported to be a common complication.^3)^ Although there was no microbiological evidence, an infected ACL seems to be the most likely cause for the fevers and significantly elevated inflammatory markers of our patient. The patient had been treated with antibiotics for several days prior to the surgery, which could have prevented bacterial growth in the culture medium.

The typical features of ACL on ultrasound imaging show a thin-walled, fluid-filled cystic structure with clear boundaries and no blood flow in color Doppler imaging. In some cases, solid echogenicity with a honeycomb pattern or cobweb appearance can also be detected. For more complex, multi-locular ACL, a CT scan can provide more information regarding the extent of the disease, with typical ACL presenting as low-density cysts with an average of 19.7 Hounsfield units with a thin, regular-shaped cystic wall, homogenous cystic content, and no contrast enhancement.^7,15)^

However, in our case, the CT scan showed large amounts of loculated ascites with dot-like calcifications, encasement of bowel loops, rectum, and ovaries without evidence of invasion, displacement of the small bowel and the normal mesenteric fat, as well as scalloping of the liver surface—radiological features that have been described as highly specific for the diagnosis of pseudomyxoma peritonei.^16,17)^ The intraoperative findings were thought to be a further testament to PMP as archetypical findings with abundant mucinous ascites, cystic implants on peritoneal surfaces, an omental cake, and ovarian involvement were observed.^12)^

Nevertheless, cytoreductive surgery and hyperthermic intraperitoneal chemotherapy (CRS/HIPEC) are considered the gold standard in the treatment of PMP. In ACL, however, complete surgical resection is the treatment of choice. In retrospect, the HIPEC component of our treatment strategy was not indicated. A preoperative tissue diagnosis could have prevented this potentially harmful aspect of the treatment, although it was well tolerated by our patient. A percutaneous biopsy was deemed unnecessary due to the archetypical clinical and radiological findings for PMP. Furthermore, the diagnostic value of preoperative percutaneous biopsies is very limited in the case of PMP and often reveals acellular mucin only when obtained in the setting of a (postoperatively) histologically proven PMP and a mucinous/gelatinous appearance has also been described for ACL.^12,18,19)^ Moreover, biopsy results in ACL and PMP—as well as the results of intraoperative rapid pathological examinations—often remain inconclusive, and the final diagnosis of these entities is regularly made by histopathological examination of the whole specimen.^8,20–22)^

Notwithstanding this, a correct diagnosis of ACL, which may have been achieved by a preoperative percutaneous biopsy or an intraoperative rapid pathological examination, would have resulted in a more organ-sparing approach, and no HIPEC would have been performed.^23)^ Mitomycin C, as a cytotoxic agent, has various potentially harmful side effects, with neutropenia being the most common.^7,24)^

In areas with a high incidence of tuberculosis, the cavitatory form of mesenteric lymph node tuberculosis is another important differential diagnosis of ACL, with several cases being treated as tuberculosis before the final diagnosis of ACL is made by histopathology.^6)^ Furthermore, mucinous cystadenomas, mesenteric cysts, lymphomas, duplication cysts, ovarian cysts, and—in endemic regions—hydatid cysts should also be taken into consideration after peritoneal carcinomatosis of an invasive cancer has been ruled out.^3)^

## CONCLUSIONS

ACL is a very rare disease and even more uncommon in the adult population. The correct diagnosis is rarely made prior to surgery. A complete surgical resection, however, is the gold standard for symptomatic ACL. Differential diagnoses such as mesenteric cysts, mucinous cystadenomas, abdominal tuberculosis, and PMP need to be taken into consideration.

## DECLARATIONS

### Funding

This research received no specific grant from any funding agency in the public, commercial, or not-for-profit sectors.

### Authors' contributions

Andreas R. R. Weiss drafted and wrote the manuscript. Georg F. Weber, Maximilian Brunner, Robert Grützmann, Abbas Agaimy, and Christian Krautz critically revised the manuscript.

All authors approved the final version of the article.

All authors agreed to be accountable for all aspects of the work.

### Availability of data and materials

Not applicable.

### Ethics approval and consent to participate

This case report was exempt from the Institutional Review Board standards at the Friedrich-Alexander-University Erlangen-Nürnberg (FAU).

### Consent for publication

The patient involved in this study gave written informed consent authorizing the use and disclosure of her protected health information.

### Competing interests

The authors declare no competing interests.
